# Factors associated with health-related quality of life in a working population in Singapore

**DOI:** 10.4178/epih.e2020048

**Published:** 2020-06-30

**Authors:** Dhiya Mahirah, Charlotte Sauter, Thuan-Quoc Thach, Gerard Dunleavy, Nuraini Nazeha, George I. Christopoulos, Chee Kiong Soh, Josip Car

**Affiliations:** 1 Centre for Population Health Sciences, Lee Kong Chian School of Medicine, Nanyang Technological University, Singapore; 2 Division of Strategy, Management and Organisation, Nanyang Business School, College of Business, Nanyang Technological University, Singapore; 3 School of Civil and Environmental Engineering, College of Engineering, Nanyang Technological University, Singapore

**Keywords:** Quality of life, Occupational health, Workplace, Singapore

## Abstract

**OBJECTIVES:**

This study aimed to evaluate the determinants of health-related quality of life (HRQoL) among workers in Singapore.

**METHODS:**

We analysed data from a cross-sectional study of 464 participants from 4 companies in Singapore. Physical and mental components of HRQoL were assessed using the Short-Form 36 version 2.0 survey. A generalized linear model was used to determine factors associated with the physical component summary (PCS) and mental component summary (MCS) scores of HRQoL.

**RESULTS:**

The overall mean PCS and MCS scores were mean±standard deviation 51.6±6.7 and 50.2±7.7, respectively. The scores for subscales ranged from 62.7±14.7 for vitality to 83.5±20.0 for role limitation due to emotional problems. Ethnicity, overweight/obesity, and years working at the company were significantly associated with physical HRQoL, and age and stress at work were significantly associated with mental HRQoL. Moreover, sleep quality was significantly associated with both physical and mental HRQoL.

**CONCLUSIONS:**

These findings could help workplaces in planning strategies and initiatives for employees to maintain a worklife balance that encompasses their physical, emotional, and social well-being.

## INTRODUCTION

Health-related quality of life (HRQoL) is a measurement of quality of life that evaluates physical, mental, and social dimensions of health [1]. It expands upon clinical measurements and provides a reliable tool to measure perceptions and subjective experiences of health [2,3]. HRQoL research serves to identify the needs of a population. Hence, it can inform policy-makers on the potential impact of interventions for disease prevention and treatment on physical and mental health [4].

Adults spend a significant amount of time at their workplace; therefore, the working environment has a substantial impact on people’s mental and physical well-being [5]. Previous research showed that job strain [6], lack of social support, and work-related stress were associated with lower levels of HRQoL [7,8]. The effect of work-related stress on mental and physical health varies by age group, sex, and education [5]. The overall perception of HRQoL can also be determined by individual-level lifestyle factors, such as smoking and drinking alcohol [9], physical activity [10], and obesity [11]. Moreover, different occupational groups vary in terms of the physical and mental demands associated with the job scope, which may have different health implications [12,13]. For instance, working night shifts may lead to negative health effects as compared to working day shifts [14] and healthcare workers may report greater burnout than non-health care workers [15].

Singapore’s fast-growing economy is characterized by increased professionalization and a focus on productivity and competition, which puts pressure on workers to perform and generate output [13]. Consequently, workers’ well-being or HRQoL may be compromised, which is associated with lower productivity. This may result in an economic threat or major costs for the company [5]. Moreover, the employment rate in Singapore has been growing over the past decade, and it is expected that an increasing proportion of the older population will remain employed, due to a rise of the re-employment age to 67 years (and eventually to 70 years in 2030) [16]. Hence, to cope with the increasing demand from the economy and the aging workforce, promoting work-life balance would lead to higher job satisfaction and better mental well-being, thereby overcoming any losses due to better worker welfare [17].

Prior studies of HRQoL in Singapore have focused on the general population [2], elderly individuals [18], or specific health indicators like obesity [11]. In view of the significant impact work has throughout one’s lifetime and the variability in health effects observed across different occupational groups, it is worthwhile to investigate HRQoL from an occupational perspective. This study provides findings from a multi-ethnic working population from various occupational backgrounds, such as engineers, technicians, university faculty, and administrative staff. It also took into consideration a combination of workplace factors, such as workplace location (aboveground vs. underground), occupation type (desk job vs. non-desk job) and duration of employment with the company, which were not assessed in earlier studies. Hence, we aimed to identify determinants influencing the physical and mental well-being of individuals of diverse ethnicities in a workplace environment.

## MATERIALS AND METHODS

### Study design

We conducted a cross-sectional analysis of the baseline data of a workplace cohort study in Singapore. Details of the cohort study design have been reported elsewhere [19]. Briefly, the study comprised 464 full-time employees from 4 companies. Employees from the transport industry, cooling plants, and universities were recruited via workplace posters and emails. They were eligible for participation if they fulfilled the following criteria: at least 21 years old, English-speaking, working for at least 4 hours per day, and not pregnant. Recruitment and baseline assessment were conducted from August 2017 to March 2018.

#### Outcome variable

HRQoL was assessed using the 36-Item Short-Form Survey version 2.0 (SF-36v2) [20]. The SF-36v2 is a well-established, validated, and standardised instrument that covers 8 health subscales: physical functioning (10 items), role limitation associated with physical problems (4 items), role limitation associated with emotional problems (3 items), bodily pain (2 items), general health (5 items), mental health (5 items), social functioning (2 items), and vitality (4 items). These subscales are aggregated into 2 summary scores, the physical component summary (PCS) and the mental component summary (MCS) [3]. The summary scores are derived from a linear t-score transformation to produce a mean of 50 and standard deviation of 10, normed to the United States general population. Scores for each subscale and component range from 0 to 100, with higher scores reflecting better quality of life. In our study, PCS and MCS scores were used as a measure of HRQoL.

#### Socio-demographic factors

Data on age group (21-30, 31-40, and > 40 years), sex (male and female), ethnicity (Chinese, Malay, Indian, and others), educational level (primary and secondary, pre-college and college and above) and average monthly income (< US$2,890 and ≥ US$2,890) were collected using self-administered questionnaires. Income categories were based on the approximate median gross monthly income in Singapore (SG$4,056= US$2,890) [21].

#### Health and lifestyle behaviours

Information on smoking status (non-smoker or current smoker), frequency of alcohol consumption (non-drinker, less than once a month, or more than once a month) and self-reported comorbidities (no morbidity or at least 1 morbidity) were collected using a self-administered questionnaire. Anthropometric measurements of height and weight were measured in accordance with the World Health Organization STEPwise approach to surveillance (WHO STEPS) protocol [22]. Body mass index (BMI) was calculated as weight in kilograms divided by the square of height in meters (kg/m^2^). Overweight/obese categories (no: BMI < 23 kg/m^2^, yes: BMI ≥ 23 kg/m^2^) were defined according to the WHO Asian BMI cut-off [23].

The Global Physical Activity Questionnaire was used to measure physical activity in the domains of work, transport, and leisure [24]. Metabolic equivalent (MET) values of 4 and 8 were assigned to moderate-intensity and vigorous-intensity activities, respectively. The overall value for MET-min/wk was calculated by adding the product of the total time (in minutes) spent in physical activity by its MET values for work, transport, and leisure domains. Low, moderate, and high physical activity levels were defined as engaging in < 600, 600-2,999, and ≥ 3,000 MET-min/wk, respectively [22]. Information on sedentary behaviour was also obtained by estimating the time spent (in hours) sitting or reclining in a day.

The 19-item Pittsburgh Sleep Quality Index (PSQI) was used to assess sleep quality [25]. The PSQI consists of 7 components rated on a scale of 0-3, and a global score was obtained by summing up the scores according to the standard PSQI scoring method. Higher scores of the PSQI indicate poorer sleep quality.

#### Workplace factors

Information on shift work (yes or no), workspace location (aboveground or underground), occupation type (desk job or non-desk job), frequency of stress experienced at work (never, some, and several periods or permanent stress), hours spent at work per day and number of years employed in the current company were also obtained. In our study, employees with desk occupation type were generally desk-bound and based in office spaces or control rooms. Non-desk occupation type employees were based in workshops. They were mostly involved in repair work and had no fixed workstations or desks. An underground workspace location was defined as being below street level and without access to windows with natural light from their workstation.

### Statistical analysis

We summarized continuous variables as mean±standard deviation, and categorical variables as frequency and percentage. Continuous data were checked for skewness, and skewed variables were presented as median and interquartile range. To identify factors associated with PCS and MCS, a generalized linear model was fitted with a normal distribution and an identity link function using a robust standard error in the following hierarchical fashion:

Model 1: included socio-demographic characteristics; Model 2: retained variables of model 1 with a p-value < 0.20 and included health factors/lifestyle behaviours; Model 3: retained variables of model 2 with a p-value < 0.20 and included occupational factors; Model 4 (final model): retained variables of model 3 with a p-value < 0.20.

A two-tailed p-value < 0.05 was considered to indicate statistical significance. All statistical analyses were conducted using Stata version 15.0 (StataCorp., College Station, TX, USA).

### Ethics statement

The study was conducted in accordance with the Declaration of Helsinki, and approved by the Institutional Review Board (IRB) of Nanyang Technological University (NTU) (IRB-2015-11-028). The study participants provided written informed consent prior to the commencement of data collection.

## RESULTS

Table 1 summarizes the characteristics of the study participants. The mean age of the participants was 39.0±11.4 years and two-thirds (63.8%) were Chinese. The majority of the participants were male (79.5%) and more than one-third (35.6%) had a college degree or higher. Most participants (78.7%) were desk-based workers and almost one-third were shift workers (35.8%).

Table 2 presents a summary of scores for the physical and mental HRQoL components and the 8 subscales of the SF-36v2. The overall mean PCS and MCS scores were 51.6±6.7 and 50.2±7.7, respectively. With regards to the subscales, role limitation as a result of emotional problems had the highest score (83.5±20.0), whereas vitality had the lowest (62.7±14.7) in the study population. Differences in the mean scores of PCS, MCS, and the 8 subscales by socio-demographic characteristics are presented in Supplementary Material 1.

Table 3 shows the bivariate associations between covariates with PCS and MCS scores. Sex, ethnicity, education, average monthly income, overweight status, sedentary behaviour, smoking status, alcohol drinking, sleep quality, and occupation type were all significantly associated with PCS scores. Employees who were female (compared to males), had a pre-college education or higher (compared to primary and secondary education), drank more than once a month (compared to non-drinkers), and worked desk-based jobs (compared to non-desk-based jobs) showed significant univariate associations with higher PCS scores. In contrast, Malay employees (compared to Chinese), overweight/obese employees (compared to not overweight), and current smokers (compared to non-smokers) had significantly lower PCS scores. With respect to MCS scores, employees aged 41 years or older reported higher scores than their younger counterparts. Lower sleep quality scores were significantly associated with lower PCS and MCS scores.

Table 4 presents predictors of PCS and MCS scores. Of particular interest, variables significantly related to PCS scores in the final model (model 4) were ethnicity, overweight or obesity, sleep quality, and years at the company. Malay ethnicity (compared to Chinese ethnicity), being overweight or obese, and worse sleep quality were significantly associated with lower PCS scores. Participants who were employed in the company for a longer period tended to have higher PCS scores, while being a member of the age group ≥ 41 years was associated with higher MCS scores than the 21-30 years group. Employees with worse sleep quality and those experiencing some or permanent stress (vs. no stress) at work were significantly more likely to have lower MCS scores.

## DISCUSSION

The present study explored the possible associations of sociodemographic characteristics and health/lifestyle factors with work-related factors on HRQoL, as measured by the validated SF36v2 questionnaire. The mean scores of the physical (PCS) and mental (MCS) components of HRQoL for our working population are comparable to the national norms established in a national cohort study within the Singaporean population (PCS, 50.0; MCS, 49.6) [2]. Our study found that ethnicity, overweight/obesity, and years at the company were significantly associated with physical HRQoL. Age and stress at work were significantly associated with mental HRQoL. Sleep quality was significantly associated with both the physical and mental aspects of quality of life.

### Socio-demographic risk factors

We found that age and ethnicity were significantly associated with MCS and PCS, respectively. Older workers reported higher MCS scores, which is consistent with the findings of other studies that older age is associated with better mental health outcomes [26]. Workers in the older age group benefit from better mental health when highly engaged in their work [27]. In an earlier longitudinal study conducted on middle-aged and older Singaporeans, work engagement was shown to promote improved mental well-being, as employment provides opportunities for social interactions and cognitive engagement [28]. In contrast to the younger age group, older workers may have built up resilience in managing changes or stressors in the work environment from their years of experience in the workplace, enabling them to enjoy better mental well-being [27].

We also observed that Malay workers were more likely to report lower PCS scores than their Chinese counterparts. In a national cohort study conducted in Singapore, participants of Chinese ethnicity reported significantly higher PCS than those of other ethnic groups (i.e., Malay and Indian). However, in that study, the effect of ethnicity on physical HRQoL in PCS was no longer significant after adjusting for other variables. Previous studies in Singapore have reported ethnic differences in some health conditions and behaviours. For instance, individuals of non-Chinese ethnicity were more likely to experience migraines [29] and type 2 diabetes [30], meaning that they are more likely to report poorer physical well-being. There may be cultural differences in help-seeking behaviours amongst the different ethnicities, especially in the way they perceive physical symptoms and seek professional help for diagnosis or treatment [31]. For instance, Malays may perceive pain conditions as involving spiritual resilience, whilst Chinese may seek alternative treatments like acupuncture or traditional Chinese medicine to treat their symptoms. A broad range of factors mediate relationships between ethnicity and health, such as cultural beliefs and behaviour, social networks, family stress, and personality characteristics [32]. Thus, it is crucial to note that the use of PCS as a measure of physical HRQoL may not be sensitive to the complex nature of ethnic or cultural differences in the current population.

### Health and lifestyle-related factors

Of all the lifestyle and health factors measured, only overweight or obesity significantly contributed to physical HRQoL in our working population. This is aligned with previous research in Singapore that has identified associations between a higher BMI and lower physical HRQoL [11]. Further studies have also supported associations between obesity and the development of musculoskeletal disorders, which result in back, joint, or overall muscle pain that may impede employees’ ability to perform work-related physical tasks [33]. Whilst it is expected that participants who were overweight/obese would report lower scores on physical HRQoL, it is also worth exploring other aspects of their behaviour that may contribute to their lower perceptions of physical well-being. Notably, treatment-seeking overweight or obese individuals are more likely to report lower HRQoL, in the domains of bodily pain, general health, and vitality, than their non-treatment seeking counterparts [34]. Their lower perceptions of well-being could be a result of greater body dissatisfaction and more effort should be made to promote healthy lifestyles to support a positive body image for these individuals.

In our current sample, workers who were overweight or obese reported significantly lower scores on subscales measuring physical functioning and physical role limitation (data not shown). Similarly, a workplace study found that employees in the higher BMI categories (≥ 35 kg/m^2^) were observed to experience significantly greater difficulty in performing work-related physical tasks [35]. This was estimated to result in a 1.2% health-related loss in productivity as compared to employees in the lower BMI categories. In general, productivity loss as a result of work limitations in overweight or obese workers could be attributed to increased absenteeism, which refers to the inability to work, or increased presenteeism (i.e., the inability to work at full capacity, while being present on the job) [36].

Consistent with extant research, worse sleep quality was significantly associated with poorer physical and mental HRQoL. Studies of people with insomnia using the 36-Item Short-Form Survey have consistently reported impairments in diverse aspects of HRQoL, when compared to those with better sleep quality [37]. The association between these 2 variables is consistent despite differences in the classifications and severity of insomnia or poor sleep. There are numerous health consequences of the lack of good sleep, involving both the physical and emotional aspects of functioning [38]. Shorter sleep duration with sleep disturbances was previously identified to have an effect on coronary heart disease, and shorter sleep duration was associated with an increased risk of cardiovascular complications, including stroke [39]. Sleep disturbance and poor sleep quality can, amongst other effects, result in decreased motivation and attention deployment and overall lower psychosocial well-being [40]. This is also relevant to performance at work, as poor sleep quality is associated with concentration and organization. A stress-health model describing the inter-relationships of sleep, stress, and illness incorporates both poor sleep and stress as contributors to allostatic load, which refers to the repeated exposure and adaptation to stress that results in physiological strain [41]. This would result in illness, which in turn contributes to stress and poor sleep, thereby perpetuating a cycle of poor sleep and declining mental and physical health.

### Work-related factors

Workers who experienced varying degrees of stress at work in our study reported significantly poorer mental HRQoL. According to a recent well-being survey, 92% of working Singaporeans reported being stressed at work, which is well above the global average of 84% [42]. With Singapore’s rapid industrialisation and economic growth, the pressure to meet higher expectations for productivity and efficiency is inevitable. Other related studies on working professionals found strong negative associations between occupational stress and HRQoL [5]. Workers with lower work autonomy and control might perceive higher levels of stress, which is often observed amongst less professionalized jobs [13]. A person’s natural response to stress is multidimensional. Physiologically, activation of the autonomic nervous system, consisting of the hypothalamic-pituitary-adrenal and sympatho-adrenomedulllary axes, makes the body vulnerable to diseases [43]. At the cognitive level, to cope with the combination of high demands and low control, an individual may maintain a state of high sustained effort, known as strain [44]. Increased job strain has been shown to have substantial negative influences on workers’ mental health, in addition to the loss of productivity from not being able to work at optimum conditions [6].

In our study, the number of years spent at the company was significantly associated with physical HRQoL, but not mental HRQoL. When adjusted for other variables, the association remained significant (p< 0.05), with more years at the company-related to better physical HRQoL. We hypothesized that employees working for a longer period at their current company are better at adjusting and adapting to changes in the workplace, and are therefore more resilient and may perceive better physical well-being. In a previous study of morbidly obese patients, being employed was a protective factor for physical and mental HRQoL. This was most marked in the subscales of physical functioning, physical role limitation, bodily pain, and emotional role limitation [45]. The social networks formed in the workplace, as well as being physically and mentally occupied at work, contribute to the positive observed associations of HRQoL.

The outcome of HRQoL may differ between occupational groups, as mental and physical characteristics vary, and non work-related factors (e.g., level of education or lifestyle) differ between groups [12]. In our study, occupation type was significantly associated with physical HRQoL, with desk job workers reporting higher PCS scores than those with non-desk jobs. However, this was no longer significant after adjustment for other variables, which could be due to the associations between occupation type and lifestyle or health-related behaviours such as physical activity or smoking status.

In light of the extent to which overweight/obesity is significantly associated with workers’ physical HRQoL, a health promotion programme initiated at the workplace, where individuals spend much of their time, may benefit the most for these workers. Health promotion programmes at the workplace ensure that a significant proportion of the adult population is reached within a relatively controlled setting. Weight loss programmes at the workplace have been shown to improve physical HRQoL, particularly in the domains of physical functioning, physical role limitation, and general health perceptions [46]. For such programmes to be effective, they need to be accessible and easily adhered to, meaning that they must be low-cost and provide strong socio-emotional support for participants. Workplace policies and strategies should also be aimed at promoting work-life balance and managing the emotional and physical responses to specific job demands so as to ensure the holistic (i.e., physical, mental, and social) well-being of employees. A holistic approach enables programmes to address workers’ experience of stress at work with the aim of improving their overall mental HRQoL. For instance, workplaces could focus on building a supportive management system, reducing unfavourable work conditions and promoting growth, autonomy, and opportunities for employees [47]. This would help to build resilience in workers and equip them with strategies to cope with stressors in the work environment, thus improving their perceptions of their functional ability and general well-being. For employers, the possibility of increased productivity could be a strong incentive to embark on such initiatives.

### Strengths and limitations

Some strengths of the current study include the use of a large and representative sample of workers from different occupations and work environments. We also included an extensive list of health, lifestyle, and work-related variables that have been related to poor health outcomes in working populations, and there were little missing data for the variables measured.

There are also limitations present in this study. The cross-sectional study design does not allow for the examination of causality between the independent variables and HRQoL. Moreover, reverse causality cannot be ruled out. Longitudinal data would be necessary to understand the direction of causality. There is also a possibility of self-selection bias in the recruitment of participants. Employees who are experiencing poorer HRQoL may have been more motivated to participate in the study than those who have better HRQoL. Therefore, the observed associations may not be generalisable to the wider working population in Singapore. Although the results are based on different occupation types, the group of non-desk job workers was too small for a stratified analysis to identify differences in risk factors for different occupation types. In addition, physical and mental HRQoL were assessed by a self-reported questionnaire. Assessing physical and mental HRQoL with validated and culturally adopted tools may be necessary [48]. This could provide better insights into the well-being or quality of life of the working population. Furthermore, there might be additional variables that were not adjusted in the analysis. We assessed stress at work, but other work-related factors (e.g., decision latitude, job demands, or social support) have been shown to be related to HRQoL [49]. For workspace location, we tested aboveground versus underground locations. However, there are other factors in the working environment, such as workspace design and functional comfort, that could also affect workers’ quality of life [50].

## Figures and Tables

**Table 1.** Characteristics of the study participants (n=464)

Characteristics	Mean±SD or n (%)
Socio-demographic factors	
Age (yr)	39.0±11.4
21-30	153 (33.0)
31-40	121 (26.1)
≥41	190 (41.0)
Sex	
Male	369 (79.5)
Female	95 (20.5)
Ethnicity	
Chinese	296 (63.8)
Malay	99 (21.4)
Indian	48 (10.3)
Others^1^	21 (4.5)
Education	
Primary and secondary	116 (25.0)
Pre-college	183 (39.4)
College and above	165 (35.6)
Average monthly income (US$)	
<2,890	331 (71.3)
≥2,890	133 (28.7)
Health/lifestyle factors	
Overweight/obesity (BMI ≥23 kg/m^2^)	
No	153 (33.0)
Yes	311 (67.0)
Comorbidities	
No	295 (63.6)
≥1	169 (36.4)
Physical activity (MET-min/wk)	
Low (<600)	107 (23.1)
Moderate (600-2,999)	200 (43.1)
High (≥3,000)	157 (33.8)
Sedentary behaviour (hr)	6.7±3.7
Smoking status	
Non-smoker	303 (65.3)
Current smoker	161 (34.7)
Alcohol drinking	
Non-drinker	216 (46.5)
Less than once a month	161 (34.7)
More than once a month	87 (18.8)
Sleep quality – PSQI score	5.5±2.8
Work-related factors	
Shift work	
No	298 (64.2)
Yes	166 (35.8)
Work location	
Aboveground	322 (69.4)
Underground	142 (30.6)
Occupation type	
Desk job	365 (78.7)
Non-desk job	99 (21.3)
Stress at work	
Never	80 (17.3)
Some	279 (60.1)
Several periods/permanent stress	105 (22.6)
Hours at work	8.6±1.3
Years at company^2^	3.0 [1.0, 7.0)]

SD, standard deviation; BMI, body mass index; MET, metabolic equivalent task; PSQI, Pittsburgh Sleep Quality Index.

1 Includes mixed ethnicities, Indonesians, and Filipinos.

2 Median [interquartile range] reported for non-normally distributed variables.

**Table 2.** Mean and SD of SF-36v2 subcategory scores

Variables	Mean±SD
Physical component summary	51.6±6.7
Physical functioning	80.9±24.4
Role physical	82.7±20.0
Bodily pain	76.8±19.2
General health perceptions	67.4±16.8
Mental component summary	50.2±7.7
Vitality	62.7±14.7
Social functioning	81.4±18.7
Role emotional	83.5±20.0
Mental health	74.7±14.2

SD, standard deviation; SF-36v2, 36-item Short-Form Survey version 2.0.

**Table 3.** Univariate analysis between covariates with PCS and MCS^1^

Characteristics	PCS	MCS
Socio-demographic factors		
Age (yr)		
21-30	Reference	Reference
31-40	-1.40 (0.090)	0.85 (0.379)
≥41	-0.59 (0.408)	2.43 (0.002)
Sex		
Male	Reference	Reference
Female	1.43 (0.032)	0.83 (0.301)
Ethnicity		
Chinese	Reference	Reference
Malay	-5.01 (<0.001)	-0.79 (0.397)
Indian	-1.70 (0.086)	1.71 (0.092)
Others^1^	-0.05 (0.675)	2.13 (0.191)
Education		
Primary and secondary	Reference	Reference
Pre-college	2.05 (0.017)	0.62 (0.496)
College and above	4.36 (<0.001)	-0.11 (0.911)
Average monthly income (US$)		
<2,890	Reference	Reference
≥2,890	2.08 (0.001)	-0.23 (0.790)
Health/lifestyle factors		
Overweight/obesity		
No	Reference	Reference
Yes	-2.92 (<0.001)	0.52 (0.476)
Comorbidities		
No	Reference	Reference
≥1	-0.78 (0.223)	-1.42 (0.073)
Physical activity (MET-min/wk)		
Low (<600)	Reference	Reference
Moderate (600-2,999)	0.16 (0.835)	0.75 (0.396)
High (≥3,000)	-0.68 (0.428)	0.90 (0.356)
Sedentary behaviour (hr)	0.28 (0.001)	-0.19 (0.064)
Smoking status		
Non-smoker	Reference	Reference
Current smoker	-2.24 (0.001)	-1.14 (0.148)
Alcohol drinking		
Non-drinker	Reference	Reference
Less than once a month	1.28 (0.070)	-0.99 (0.210)
More than once a month	2.16 (0.005)	-1.47 (0.152)
Sleep quality - PSQI score	-0.67 (<0.001)	-0.96 (<0.001)
Work-related factors		
Shift work		
No	Reference	Reference
Yes	-0.46 (0.479)	-1.19 (0.117)
Work location		
Aboveground	Reference	Reference
Underground	0.02 (0.977)	-0.95 (0.226)
Occupation type		
Non-desk job	Reference	Reference
Desk job	3.61 (<0.001)	0.69 (0.434)
Stress at work		
Never	Reference	Reference
Some	0.40 (0.654)	-4.54 (<0.001)
Several periods/permanent stress	1.15 (0.279)	-9.50 (<0.001)
Hours at work	0.43 (0.051)	-0.41 (0.122)
Years at company	0.08 (0.004)	0.05 (0.164)

Values are presented as coefficient (p-value). PCS, physical component summary; MCS, mental component summary; BMI, body mass index; MET, metabolic equivalent task; PSQI, Pittsburgh Sleep Quality Index.

1 From a generalized linear model.

2 Includes mixed ethnicities, Indonesians, and Filipinos.

**Table 4.** Results of generalized linear models for the SF-36v2 PCS and MCS (n=464)

Characteristics			Model 1		Model 2		Model 3		Model 4	
			PCS	MCS	PCS	MCS	PCS	MCS	PCS	MCS
Socio-demographic factors										
	Age (yr)									
		21-30	Reference	Reference	Reference	Reference	Reference	Reference	Reference	Reference (0.001)^2^
		31-40	-1.66 (0.031)	1.01 (0.283)	-0.94 (0.201)	1.02 (0.248)	-1.35 (0.071)	1.43 (0.105)	-1.20 (0.099)	1.48 (0.081)
		≥41	-0.66 (0.425)	2.97 (0.001)	0.29 (0.709)	2.93 (0.001)	-1.13 (0.204)	3.14 (0.001)	-0.69 (0.426)	3.02 (<0.001)
	Sex									
		Male	Reference	Reference	Reference	Reference	Reference	Reference	Reference	Reference
		Female	0.30 (0.654)	0.96 (0.242)	-0.40 (0.545)	0.86 (0.285)	-0.60 (0.398)	0.43 (0.598)	-0.74 (0.256)	0.43 (0.590)
	Ethnicity									
		Chinese	Reference	Reference	Reference	Reference	Reference	Reference	Reference (0.006)^2^	Reference (0.111)^2^
		Malay	-3.95 (<0.001)	-0.07 (0.945)	-3.55 (<0.001)	-0.53 (0.635)	-3.12 (0.001)	-0.73 (0.527)	-3.22 (0.001)	-0.62 (0.578)
		Indian	-1.27 (0.210)	1.91 (0.080)	-1.54 (0.121)	1.25 (0.263)	-1.27 (0.213)	0.43 (0.693)	-1.25 (0.203)	0.51 (0.642)
		Others^2^	-0.40 (0.737)	2.52 (0.101)	0.06 (0.958)	2.99 (0.047)	0.52 (0.635)	2.85 (0.029)	0.37 (0.735)	2.90 (0.028)
	Education									
		Primary and secondary	Reference	Reference	Reference	Reference	Reference	Reference	Reference (0.070)^2^	Reference
		Pre-college	1.26 (0.134)	0.90 (0.344)	1.32 (0.085)	1.32 (0.139)	1.19 (0.141)	1.63 (0.082)	1.14 (0.137)	1.67 (0.074)
		College and above	2.63 (0.009)	0.97 (0.374)	2.18 (0.014)	0.57 (0.591)	2.13 (0.036)	1.04 (0.346)	2.13 (0.021)	1.12 (0.295)
	Average monthly income (US$)									
		<2,890	Reference	Reference	-	-	-	-	-	-
		≥2,890	0.54 (0.446)	-0.98 (0.269)	-	-	-	-	-	-
Health/lifestyle factors										
	Overweight/obesity									
		No	-	-	Reference	Reference	Reference	Reference	Reference	-
		Yes	-	-	-1.40 (0.019)	1.06 (0.151)	-1.19 (0.049)	0.47 (0.513)	-1.43 (0.015)	-
	Comorbidities									
		No	-	-	Reference	Reference	-	Reference	-	Reference
		≥1	-	-	-0.29 (0.640)	-1.26 (0.106)	-	-1.03 (0.170)	-	-0.98 (0.189)
	Physical activity (MET-min/wk)									
		Low (<600)	-	-	Reference	Reference	Reference	Reference	Reference	Reference
		Moderate (600-2,999)	-	-	0.98 (0.177)	1.21 (0.145)	0.86 (0.254)	1.16 (0.161)	0.78 (0.276)	1.17 (0.157)
		High (≥3,000)	-	-	1.26 (0.118)	1.18 (0.219)	1.92 (0.020)	1.33 (0.190)	1.40 (0.077)	1.37 (0.172)
	Sedentary behaviour (hr)		-	-	0.22 (0.010)	-0.04 (0.669)	0.13 (0.152)		0.16 (0.073)	
		Smoking status								
		Non-smoker	-	-	Reference	Reference	-	-	-	-
		Current smoker	-	-	-0.13 (0.845)	0.10 (0.910)	-	-	-	-
	Alcohol drinking									
		Non-drinker	-	-	Reference	Reference	Reference	Reference	Reference	Reference
		Less than once a month	-	-	-1.16 (0.101)	-1.63 (0.035)	-0.90 (0.195)	-1.27 (0.104)	-1.16 (0.094)	-1.29 (0.098)
		More than once a month	-	-	0.35 (0.639)	-2.20 (0.026)	0.20 (0.790)	-1.87 (0.068)	0.24 (0.737)	-1.93 (0.060)
	Sleep quality - PSQI score		-	-	-0.57 (<0.001)	-1.01 (<0.001)	-0.61 (<0.001)	-0.74 (<0.001)	-0.57 (<0.001)	-0.74 (<0.001)
Work-related factors										
	Shift work									
		No	-	-	-	-	Reference	Reference	-	-
		Yes	-	-	-	-	0.49 (0.497)	-0.31 (0.671)	-	-
	Work location									
		Aboveground	-	-	-	-	Reference	Reference	-	-
		Underground	-	-	-	-	-0.27 (0.671)	-0.47 (0.511)	-	-
	Occupation type									
		Desk job	-	-	-	-	Reference	Reference	Reference	Reference
		Non-desk job	-	-	-	-	1.64 (0.118)	1.68 (0.110)	1.36 (0.147)	1.62 (0.109)
	Stress at work									
		Never	-	-	-	-	Reference	Reference	-	Reference (<0.001)^2^
		Some	-	-	-	-	0.23 (0.783)	-4.02 (<0.001)	-	-4.06 (<0.001)
		Several periods/permanent stress	-	-	-	-	0.73 (0.469)	-7.79 (<0.001)	-	-7.90 (<0.001)
	Hours at work		-	-	-	-	0.15 (0.512)	-0.33 (0.178)	-	-0.38 (0.108)
	Years at company		-	-	-	-	0.10 (0.013)	-0.02 (0.711)	0.08 (0.031)	-

Values are presented as coefficient (p-value). SF-36v2, 36-item Short-Form Survey version 2.0; PCS, physical component summary score; MCS, mental component score; BMI, body mass index; MET, metabolic equivalent task; PSQI, Pittsburgh Sleep Quality Index.

1 Includes mixed ethnicities, Indonesians, and Filipinos.

2 Overall p-value for significant categorical variables with more than two levels.
