# Supplementary material for: Factors associated with health-related quality of life in a working population in Singapore
Source: Epidemiol Health. 2020 Jun 30;42:e2020048. doi: 10.4178/epih.e2020048 (PMC7871151; doi:10.4178/epih.e2020048)
Supplement: Supplementary file 1 [file epih-42-e2020048-suppl.docx]

Supplementary Material 1. Mean and SD of SF-36v2 summary and subscale scores by sociodemographic characteristics

|  | Summary Scores | | Physical subscales | | | | Mental subscales | | | |
| --- | --- | --- | --- | --- | --- | --- | --- | --- | --- | --- |
|  | Physical component summary | Mental component summary | Physical functioning | Role physical | Bodily pain | General health perceptions | Vitality | Social functioning | Role emotional | Mental Health |
| Overall | 51.6 (6.7) | 50.2 (7.7) | 80.9 (24.4) | 82.7 (20.0) | 76.8 (19.2) | 67.4 (16.8) | 62.7 (14.7) | 81.4 (18.7) | 83.5 (20.0) | 74.7 (14.2) |
| Age |  |  |  |  |  |  |  |  |  |  |
| 21-30 | 52.2 (6.5) | 49.0 (7.2) | 84.4 (24.3) | 82.8 (18.3) | 77.9 (19.4) | 64.5 (16.1) | 60.5 (14.5) | 80.8 (18.1) | 82.7 (18.4) | 73.0 (14.0) |
| 31-40 | 50.8 (7.0) | 49.8 (8.4) | 78.3 (25.9) | 80.5 (20.5) | 75.6 (19.8) | 67.5 (19.1) | 60.4 (16.0) | 79.1 (20.2) | 83.5 (21.1) | 73.8 (14.4) |
| ≥41 | 51.6 (6.6) | 51.4 (7.5) | 79.8 (23.3) | 84.1 (20.8) | 76.7 (18.7) | 69.8 (15.6) | 65.9 (13.3) | 83.2 (17.9) | 84.2 (20.7) | 76.8 (14.0) |
| Gender |  |  |  |  |  |  |  |  |  |  |
| Male | 51.3 (6.9) | 50.0 (8.0) | 79.3 (25.7) | 81.4 (20.7) | 76.7 (19.5) | 67.7 (16.4) | 62.6 (15.0) | 80.5 (19.0) | 82.3 (21.0) | 74.6 (14.6) |
| Female | 52.7 (5.5) | 50.9 (6.7) | 87.2 (17.2) | 88.0 (15.8) | 77.0 (17.9) | 66.4 (18.4) | 63.0 (13.2) | 84.7 (17.1) | 88.3 (14.8) | 75.5 (12.6) |
| Ethnicity |  |  |  |  |  |  |  |  |  |  |
| Chinese | 52.8 (5.9) | 50.1 (7.8) | 84.6 (20.6) | 86.8 (17.4) | 79.6 (17.7) | 67.1 (16.9) | 62.4 (14.6) | 83.1 (18.2) | 85.6 (18.1) | 74.7 (14.6) |
| Malay | 47.8 (7.7) | 49.3 (8.1) | 70.4 (31.6) | 71.3 (22.2) | 66.5 (21.2) | 66.8 (16.0) | 60.8 (15.2) | 74.6 (20.1) | 75.5 (24.0) | 73.3 (14.0) |
| Indian | 51.1 (6.5) | 51.8 (6.4) | 80.3 (23.4) | 79.3 (21.6) | 78.1 (17.8) | 69.8 (17.2) | 67.1 (13.2) | 81.8 (17.3) | 85.4 (19.6) | 77.1 (11.1) |
| Others^a^ | 52.3 (5.6) | 52.2 (7.3) | 79.5 (24.7) | 86.3 (17.9) | 83.3 (15.4) | 69.7 (19.2) | 65.5 (15.3) | 87.5 (13.1) | 87.3 (18.4) | 77.4 (14.7) |
| Education |  |  |  |  |  |  |  |  |  |  |
| Pri and Sec | 49.2 (8.0) | 50.0 (7.8) | 70.6 (30.2) | 75.1 (23.3) | 72.4 (22.9) | 68.6 (15.9) | 65.0 (14.3) | 77.7 (19.6) | 76.1 (23.7) | 74.2 (14.4) |
| Pre-college | 51.3 (6.0) | 50.6 (7.5) | 81.7 (22.3) | 82.8 (19.6) | 75.3 (18.8) | 66.5 (16.8) | 63.0 (14.5) | 81.4 (18.9) | 84.7 (19.0) | 75.4 (13.9) |
| College and above | 53.6 (5.8) | 49.9 (8.0) | 87.2 (19.3) | 88.0 (15.7) | 81.5 (15.5) | 67.7 (17.5) | 60.7 (14.9) | 83.9 (17.3) | 87.4 (16.8) | 74.4 (14.4) |

SD: standard deviation; SF-36v2: 36-item Short-Form Survey version 2.0
